# Case Report: A case of Joubert syndrome in twin pregnancy: MRI manifestations and literature review

**DOI:** 10.3389/fped.2026.1732875

**Published:** 2026-03-18

**Authors:** Silu Ren, Aitong Li, Jiyun Yang, Lei Zhou, Tao Lu

**Affiliations:** 1 School of Medicine, University of Electronic Science and Technology of China, Chengdu, China; 2 Sichuan Provincial Key Laboratory for Human Disease Gene Study, Center of Medical Genetics, Sichuan Academy of Medical Sciences and Sichuan Provincial People's Hospital, University of Electronic Science and Technology of China, Chengdu, China; 3 Department of Radiology, People's Hospital of Shizhu Tujia Autonomous County, Chongqing, China; 4 Department of Radiology, Sichuan Provincial People's Hospital, University of Electronic Science and Technology of China, Chengdu, China

**Keywords:** differential diagnosis, Joubert syndrome and related disorders, magnetic resonance imaging, molar tooth sign, posterior fossa malformation, prenatal diagnosis

## Abstract

**Introduction:**

Joubert syndrome (JS) is a rare autosomal recessive disorder belonging to the ciliopathies and can cause a series of neurological symptoms after birth. Prenatal diagnosis of this disease is rare, as the results from prenatal ultrasonography for JSRD are relatively nonspecific. Prenatal MRI is usually the preferred diagnostic method. On fetal MRI, it presents as a typical midbrain-hindbrain malformation characterized by the molar tooth sign (MTS). Currently, reports of prenatal MRI diagnosis for JS are rare, with no documented twin gestations. Herein, we report a case of JSRD in a twin pregnancy detected at the 25th gestational week through prenatal MRI, with a review of the etiology, imaging features, and differential diagnosis of JS.

**Patient presentation:**

A 31-year-old woman was pregnant at the 25th gestational week with fetal cerebellar vermis hypoplasia suspected from prenatal US. Fetal MRI demonstrated the characteristic MTS, which is the hallmark of JS, and strongly supported the diagnosis at our hospital. The amniotic fluid prenatal diagnosis revealed that the twins had compound heterozygous TMEM67 variants classified as Variants of Uncertain Significance (VUS). The couple finally opted to terminate the pregnancy.

**Conclusions:**

Currently, the diagnosis of JS is typically made postnatally. MRI is extremely advantageous for evaluating posterior fossa structural anomalies prenatally. In combination with genetic testing, it can provide guidance for early diagnosis and prenatal counseling.

## 1 Introduction

Joubert syndrome (JS), alternatively termed molar tooth midbrain-hindbrain malformation, is a group of autosomal recessive genetic diseases that are relatively rare congenital cranial developmental malformations. First described by Joubert in 1969 (1), JS is characterized by intellectual disability, hypotonia, ataxia, tachypnea/apnea, and abnormal eye movements, with the typical neuroimaging features of the molar tooth sign (MTS). When associated with ocular, renal, hepatic, and other organ abnormalities, this condition is referred to as Joubert syndrome and related disorders (JSRDs) (2). The terms Joubert syndrome (JS) and related disorders (JSRD) have been used to define all disorders associated with MTS in brain imaging studies (3). The incidence rate of JS and JSRD is estimated to be between 1/80,000 and 1/1,00,000 live births (4).

JS is typically diagnosed postnatally due to neurological symptoms, including hypotonia, abnormal eye movements, and tachypnea/apnea at neonatal age, with additional features such as cerebellar ataxia, cognitive impairment, intellectual disability, and various growth retardations, particularly with increasing age (2, 3, 5–7). The postnatal outcomes of children with JS are largely influenced by the presence of PF anomalies. However, accurate prenatal diagnosis of PF anomalies remains challenging (8).

Prenatal ultrasound (US) serves as the primary screening modality for detecting PF malformations since it is noninvasive, widely available, and safe (9). However, during late pregnancy, US images of the cerebellum are usually compromised because of attenuation at the bony interface of the skull and the fetal head position in the maternal pelvis (10). A further limitation of prenatal US is the difficulty in obtaining sagittal views due to fetal orientation. While three-dimensional multiplanar US may help obtain sagittal views, its spatial resolution remains inferior to that of magnetic resonance imaging (MRI), particularly for precise morphometric analysis of the PF in the mid-to-late trimester (11).

In recent decades, the use of fetal MRI has significantly improved the accuracy of the diagnosis of congenital abnormalities. Owing to its superior spatial resolution and tissue contrast, fetal MRI is increasingly being considered a valuable diagnostic technique to confirm, correct, or complement questionable US findings. It also plays an essential role in evaluating fetuses with suspected US findings and/or positive family history (11, 12). However, fetal MRI before 18 weeks of gestation typically offers no diagnostic advantage over ultrasonography (13), making it more suitable for later in the second or third trimester. Furthermore, several standardized MRI-based PF morphometric assessment protocols have been established, enabling multiparametric quantification of fetal developmental indices, including the cerebellar vermis (CV) and posterior fossa volume (PFV), with developmental trajectories referenced to estimated gestational age (EGA). These approaches are crucial for the prenatal evaluation of CNS maturation (12).

Pathognomonic MTS is defined by a peculiar appearance resembling a molar tooth secondary to thickened superior cerebellar peduncles (SCPs), cerebellar vermis (CV) hypoplasia, and a deepened interpeduncular fossa on axial images at the pontomesencephalic level. As fetal MRI provides excellent visualization of PF malformations, the typical MTS and CV hypoplasia on MRI enable the early detection of JS and facilitate subsequent genetic analysis. While JSRDs are predominantly reported postnatally, prenatal diagnoses remain rare, with no prior cases documented in twin pregnancies. Herein, we report a rare case of JS in a twin pregnancy that was diagnosed via prenatal magnetic resonance imaging (MRI) and review its distinctive imaging characteristics and differential diagnosis.​

## 2 Case presentation

**Figure 1:**
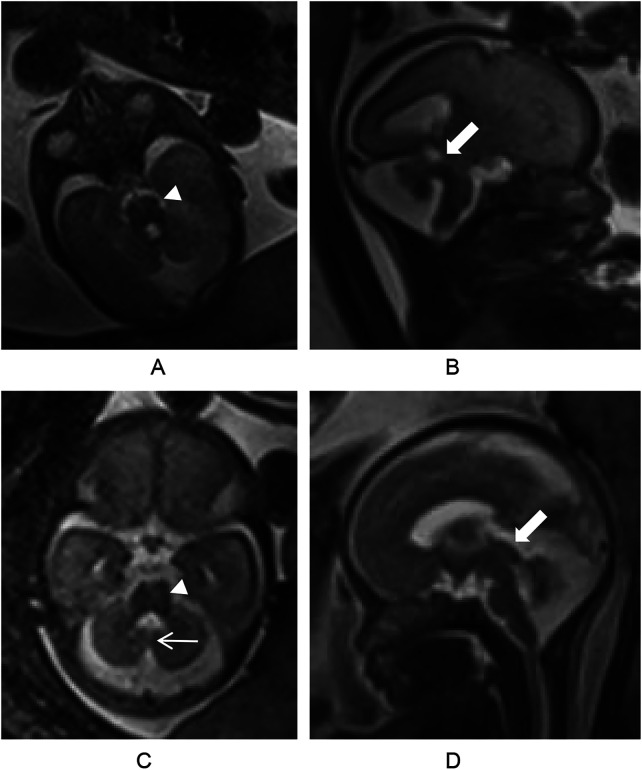
MRI of the fetuses. **(A)** Figure Axial half-Fourier acquisition single-shot turbo spin echo (HASTE) image showing the molar tooth sign of the first fetus(arrowhead). **(B)** Sagittal HASTE image showing a widened posterior fossa, cerebellar vermis dysplasia, and thickened and elongated superior cerebellar peduncles(thick arrow). The enlarged fourth ventricle communicated with the cisterna magna. **(C)** Axial HASTE image showing the molar tooth sign(arrowhead) and enlarged fourth ventricle of the second fetus,as well as the "cleft sign" (thin arrow). **(D)** Sagittal HASTE image showing cerebellar vermis dysplasia, and thickened, elongated superior cerebellar peduncles(thick arrow).

A woman, 31 years old, gravida 2, para 0, was referred to our hospital for a prenatal US consultation for suspected CV abnormality at 25 weeks gestation of a dichorionic diamniotic twin. Prenatal ultrasound revealed CV hypoplasia, and the fourth ventricle (4 V) communicated with the CM in both fetuses. US also revealed that both kidneys were enlarged and that right renal microcysts were present in both twins. Subsequent fetal MRI confirmed CV hypoplasia and typical MTS in both fetuses. The widened PF and the thickened, elongated SCPs were also demonstrated by axial half-Fourier acquisition single-shot turbo spin echo (HASTE) imaging (Figure 1). In one fetus, the enlarged 4 V also communicated with the CM.

**Figure 2:**
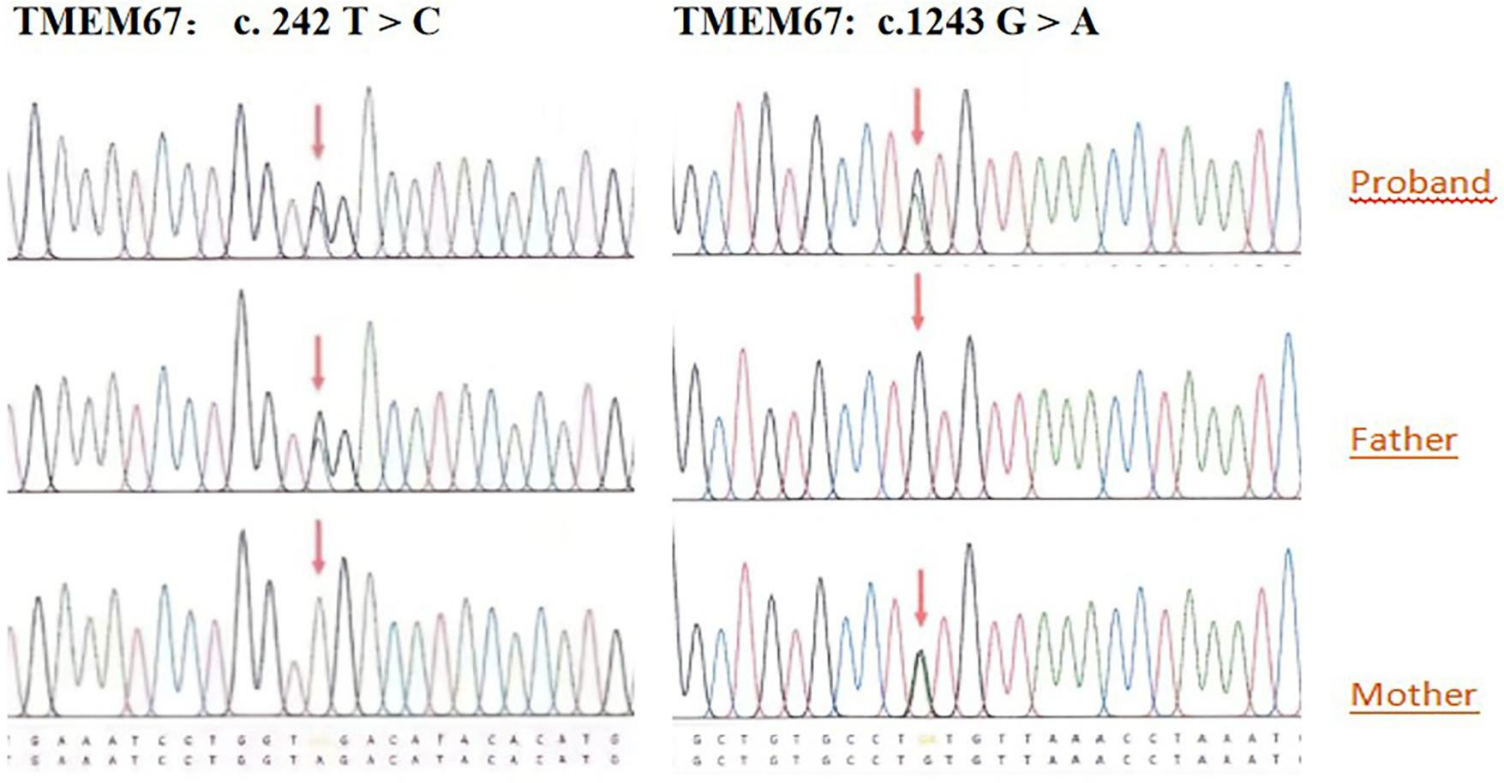
TMEM67 variant analysis in family trios. Sanger sequencing chromatograms revealed that TMEM67 (NM_153704.6), c. 242 T > C; c.1243 G > A compound heterozygous variant in the proband and carrier status in both parents.

Ultrasound-guided amniocentesis yielded fetal samples for testing. Cytogenetic analysis revealed a benign polymorphic inversion, inv(9)(p12q13), within otherwise normal karyotypes: 46,XX in Twin A and 46, XY in Twin B. Fetal sex was subsequently confirmed via STR analysis via whole-exome sequencing (WES). WES revealed compound heterozygous variants in the *TMEM67* gene (NM_153704.6) in both twins: Twin A: c.242T > C (p.Leu81Pro) [maternal] and c.1243G > A (p.Val415Met) [paternal]; Twin B: c.242T > C (p.Leu81Pro) [maternal] and c.1243G > A (p.Val415Met) [paternal] (Figure 2). According to American College of Medical Genetics and Genomics/Association for Molecular Pathology(ACMG/AMP) guidelines, both variants were classified as Variants of Uncertain Significance (VUS).

The woman had previously terminated pregnancy at 22 weeks gestation 2 years prior due to fetal polycystic kidneys, as suggested by prenatal US. However, the parents did not perform genetic testing or autopsies of the fetus. The couple was healthy and married with no consanguinity. The family members of both sides did not experience developmental delay, intellectual disability, or other nervous system abnormalities, and no other of their family members had fetuses with JS or JSRD. After the family was followed, targeted parental testing via Illumina HiSeq and Sanger sequencing confirmed their* TMEM67* carrier status. The definitive diagnosis of JS was established through integrated assessment of fetal imaging and molecular genetics. Following multidisciplinary counseling, the couple finally opted to terminate the pregnancy.

## 3 Discussion

Joubert syndrome (JS) is a distinctive cerebral neurological disorder marked by the presence of the MTS on neuroimaging. Its core clinical manifestations include intellectual disability, hypotonia, ataxia, shortness of breath/apnea, and abnormal eye movement. JS belongs to the category of ciliopathies and is closely associated with primary ciliary dysfunction. Given that primary cilia are critical for the development and function of the central nervous system as well as other organs, JS commonly involves multiple organs (3, 6). Notably, clinical data indicate that the retina is the most frequently involved organ in JSRD, followed by kidney defects (25%), polydactyly of the hands or toes (8%–16%), and hepatic fibrosis (in the minority) (3, 5, 14, 15). On the basis of the spectrum of organ involvement, scholars have classified JSRD into 6 subtypes: simple JS, JS with ocular defects (JS-O), JS with renal defects (JS-R), JS with oculo-renal defects (JS-OR), JS with hepatic defects (JS-H or COACH syndrome), and JS with oral-facial-digital defects (JS-OFD or OFD-VI syndrome). Owing to the significant phenotypic heterogeneity among patients with JSRD, formulating generalizable prognostic conclusions remains challenging (3, 6). Through a multicenter cohort study, Dempsey's team revealed that respiratory system dysfunction, chronic kidney disease, and progressive liver fibrosis are independent prognostic factors for this disease, so close surveillance for these conditions is also necessary (16).

The genetic variation in JS is diverse, and to date, more than 40 causative genes have been associated with JS. The proteins encoded by these genes are located predominantly in primary cilia or the matrix, where they play critical roles in cilial assembly and function. Among these genes, *CPLANE1*, *CC2D2A*, *AHI1*, *CEP290*, and *TMEM67* are the five major causative genes, accounting for approximately 6%–9% of JS cases (5). Fetuses carrying pathogenic variants such as *NPHP1*, *RPGRIP1L*, *TMEM231*, and *TMEM237* are typically associated with renal involvement (5). In particular, *TMEM67* variants are strongly correlated with congenital hepatic fibrosis. These mutations account for approximately 80% of liver involvement in patients with JS, representing the strongest gene‒phenotype correlation among all JS types (17). However, it is important to note that these hepatic features are more difficult to observe prenatally than the remaining phenotypes of *TMEM67*-related JS (craniocerebral, renal, and polydactyly abnormalities, etc.) (7).

In our case, heterozygous variants in the *TMEM67* gene locus were identified in both twins and their parents. Genetic analysis revealed two compound heterozygous *TMEM67* variants: c.242T > C (p.Leu81Pro) and c.1243G > A (p.Val415Met). Both were classified as Variants of Uncertain Significance (VUS) according to ACMG/AMP guidelines (18, 19). While the absence of functional assays precludes definitively classifying these variants as pathogenic under ACMG/AMP criteria, their transcompound heterozygous state provides key genetic evidence. This is strongly supported by the definitive fetal MRI finding of a molar tooth sign (MTS) and further contextualized by a prior pregnancy terminated due to fetal polycystic kidneys—a finding consistent with the *TMEM67*-related ciliopathy spectrum. The convergence of consistent genetic, imaging, and clinical data provides a compelling multi-modal basis for diagnosing *TMEM67*-related Joubert syndrome (type 6) in this case, despite the conservative VUS classification. A comprehensive integrated assessment indicates a high probability of pathogenicity for these variants.

Our genetic findings can be further elucidated by recent mechanistic insights into JS pathogenesis. A seminal 2025 study by Wang et al. (20) revealed that the Joubert syndrome 26 protein (JBTS-26) enforces compartmentalized motility of the ciliary kinesin-II through a competitive binding mechanism. This discovery provides a molecular framework for understanding how defects in diverse ciliary proteins, such as *TMEM67* identified in our case, can converge on a common pathogenic pathway—disrupting the precise spatial regulation of intraflagellar transport. The characteristic "molar tooth sign" observed on fetal MRI (Figure 1) may represent the macroscopic outcome of such microscopic disturbances in ciliary cargo delivery, which is critical for midbrain-hindbrain patterning. This precise variant interpretation is critical for accurate genetic counseling and risk assessment (21).

The *TMEM67* gene is known to be associated with a broad spectrum of disorders ranging from retinitis pigmentosa syndrome to nephronophthisis. *TMEM67* gene variants can give rise to a spectrum of phenotypes, including JS, Meckel–Gruber syndrome type 3 (MKS3), and oculo-encephalo-hepato-renal syndrome (COACH syndrome) (21). Prenatal US revealed CV hypoplasia and communication between the 4 V and CM with ARPKD-like manifestations in both fetuses, which belongs to the COACH/MKS/JS phenotype spectrum. In the context of our genetic findings, importantly, our findings highlight the necessity of comprehensive evaluation of both fetuses in a twin pregnancy when an anomaly is detected in one fetus, as these fetuses may share the same autosomal recessive condition despite potential phenotypic variances. The identification of identical *TMEM67* variants in both dizygotic twins confirms their independent inheritance from carrier parents and underscores a 25% recurrence risk for future pregnancies (22, 23). Therefore, a diagnosis based solely on US and WES is insufficient. Fetal MRI is essential for providing critical imaging evidence (e.g., MTS) for confirmation (5).

Currently, prenatal screening for fetal PF abnormalities mainly relies on US between 18 and 22 weeks of gestation. Approximately 3% of pregnant women exhibit fetal structural anomalies on routine prenatal US (19). Given that definitive prenatal genetic testing may not be conclusive in JS, the ability to identify MTS sonographically before 24 weeks can provide a valuable adjunct to prenatal diagnosis. D. Pugash (24) reported two cases in which the MTS was identified via US at 26^+4−^ and 20^+6^-week-old fetuses prior to fetal MRI or genetic testing. One fetus presented with marked CV hypoplasia, a deep cleft in the midbrain between thickened SCPs and deficiency of the dorsal midbrain in the midline, which was consistent with the MTS in the JS. The other fetus showed enlargement of the CM, CV hypoplasia, and a midline cleft of the midbrain with prominence of the SCPs on US, which was consistent with MTS. This demonstrated that even in the absence of gene variants associated with JS, US signs can help in the prenatal diagnosis of JS.

However, the precise frequency of prenatal US findings in fetuses with JS remains unclear, as the published reports described highly selected cases and applied variable criteria for CV hypoplasia/enlarged CM or failed to report all prenatal US findings across all cases (25). Furthermore, prenatal US of the PF can be technically challenging (26), and conclusive prenatal diagnosis by US remains rare. US can identify PF abnormalities but cannot provide a definitive diagnosis (24). Prenatal US findings reported in most fetuses affected with JSRD are relatively nonspecific and include an enlarged CM, CV agenesis, occipital encephalocele, ventriculomegaly, a hypoplastic phallus, renal cysts, and polydactyly. These findings can also be found in other malformations, such as Dandy‒Walker malformation and pontocerebellar hypoplasia (PCH) (7, 12, 25). Second, the US struggled to clearly identify the cerebellum and distinguish it from other structures within the PF. This limitation may result in other structures being included in the CV measurements, thus leading to a larger CV than that measured with MRI (6, 12). Additionally, the diagnostic accuracy may be compromised by insufficient US clinician familiarity with JS, potentially resulting in missed or incorrect diagnoses. Thus, prenatal imaging should search for as many phenotypes related to variants or poor prognoses (such as CV hypoplasia) as possible, especially for variants of unknown significance (7). Comprehensive phenotypic documentation is essential for improving the accuracy of both prenatal and postnatal counseling (7).

Prenatal MRI is a helpful choice, particularly in terms of the diagnosis of PF abnormalities (12, 25, 27). The advantages of MRI over US include superior tissue discrimination and resolution, elimination of artifacts, and the ability to achieve multiple planes of imaging, including sagittal views, regardless of fetal positioning, especially during the second and third trimesters (10–12). The developing calvaria can restrict US visualization of the brain, whereas this limitation does not impede visualization by MRI. In one study, the overall diagnostic accuracy for detecting an isolated posterior fossa PF abnormality by US was only 65.4%, in contrast to the significantly higher accuracy of 87.7% with fetal MRI (28). Furthermore, MRI enhances the reliability of prognostic assessments in 44% of cases, providing crucial information for prenatal counseling. Its precise quantification of lesion extent and neurofunctional impact directly influences clinical decisions in 35% of cases—including termination of pregnancy, multidisciplinary consultations, and targeted interventions (e.g., gene therapy) (28, 29). Early detection of anomalies during mid-gestation creates a window for seamless prenatal-to-postnatal management. Therefore, the clinical utility of MRI in detecting PF abnormalities lies in its ability to increase diagnostic precision via multiplanar high-resolution imaging, optimize prognostic assessment, support clinical decision-making and complement the synergistic efficacy of multimodal diagnosis.

To ensure the most accurate imaging to establish the diagnosis of MTS, MRI protocols should incorporate sequences with excellent T1 contrast as well as 3D imaging (T1- or T2-weighted) with isotropic spatial resolution, allowing reconstructions in all orientations (30) through the PF from the midbrain to the pons (31). In addition to standard axial, coronal, and sagittal slices, thin-slice axial slices are also recommended for optimal visualization (2).

Classic MTS signs include (1) bilateral SCP thickening/elongation, (2) deepened interpeduncular fossa (IF) with a narrow brainstem isthmus, and (3) CV hypoplasia, collectively forming a pathognomonic molar tooth shape on axial imaging (2, 7, 9). In the case of severe CV agenesis/hypoplasia, PF CSF spaces may communicate with the 4 V. The corresponding MRI features were the "cleft sign" in the axial and coronal planes of the cerebellum or a "bat-wing" or "umbrella-shaped" change at the top of the 4 V shown at the axial level of the midbrain, which meant that the anteroposterior diameter of the 4 V was significantly larger than its transverse diameter (32, 33). A detailed evaluation of the 4 V features in both axial and sagittal views may help in the identification of JSRD (32). The measurement of transverse cerebellar diameter, CV width and height, and CM in fetuses via MRI and comparison with published nomograms is important for detecting MTS. In fetuses demonstrating subtle or atypical MTS manifestations, it is suggested that quantitative measurements can enhance diagnostic confidence in the JSRD (32). Thus, in suspected JS, the radiological assessment should include (1) confirmation of the presence of the MTS on axial images, (2) determination of the extent of CV hypoplasia and elongation of the IF on sagittal images and (3) determination of any vermian cleft on coronal images (33).

Congenital anomalies of the CNS frequently demonstrate aberrant white matter connections. Diffusion tensor magnetic resonance imaging (DTI) and fiber tractography (FT) are powerful approaches that can indicate the orientation and integrity of white matter fibers *in vivo* (28), with fractional anisotropy (FA) used to quantitatively assess white matter microstructural integrity. Decreased FA indicates loose fiber organization or diminished myelination. In fetuses, this parameter is primarily employed to detect anomalous white matter connections associated with agenesis of the corpus callosum, gray matter heterotopia, JS, and cerebral palsy (29, 34). Studies involving adults and adolescents have shown that DTI enables visualization of the core pathological features of JS, including the abnormal decussation of the two fiber tracts, SCPs and corticospinal tracts (CST) of JS (28), irrespective of the underlying genetic variants (35). At DTI and FT, a thickened and elongated SCP with a horizontal configuration can be observed, characterized by the absence of a "focal red dot sign" in the interpeduncular fissure of the midbrain (35). Additionally, the CST fails to cross the caudal medulla. Using DTI, Charlie et al. (28) reported that the difference in the pontocerebellar tract suggested a spectrum of severity of pontine axonal migration abnormalities in 2 children with JS, especially the degree of thinning or absence of dorsal pontocerebellar (DPC) and abnormal thickening of the ventral pontocerebellar tract (VPC). These findings imply that DTI can assist in distinguishing the subtypes of JS to some extent and guiding individualized diagnostic and therapeutic strategies. Therefore, DTI-FTs demonstrate complementary findings beyond those of conventional MRI, supporting their role as a pivotal modality for both diagnosis and phenotypic stratification in patients with JS (29, 35).

In our case, prenatal US only revealed CV hypoplasia, and 4 V communicated with the CM in both fetuses. Subsequent fetal MRI confirmed typical MTS in both fetuses, further confirming the diagnosis. The genetic analysis of the amniotic fluid from the fetus, along with the genetic profiles of both parents, confirmed the variants of the *TMEM67* gene on chromosome 9, thereby clarifying the definitive diagnosis.

The differential diagnosis of JS encompasses conditions that exhibit CV dysplasia and ventricular system dilation on imaging but lack the characteristic MTS or other manifestations of systemic involvement (3, 6). The key differentials include the following (Table 1): (1) Dandy‒Walker malformation (DWM) often presents as a PF cyst with complete or partial CV agenesis/hypoplasia. On MRI, posterior cranial fossa cysts usually present as dilated 4 Vs that communicate directly with an enlarged CM, forming a continuous cystic structure distinct from the 'cleft sign' observed in JS (30). The 4 V expands posteriorly into the CM, creating a single space without SCP thickening, facilitating differentiation from the JS. While familial recurrence is rare, variant DWM requires distinction from MTS-positive JSRD cases (33). (2) Meckel-Gruber syndrome: This syndrome is characterized by occipital encephalocele, obstructive hydrocephalus, and prominent bilateral cystic renal dysplasia on prenatal MRI. Although CV hypoplasia may occur, MKS lacks the MTS. MKS results in more severe PF anomalies, which are rare in JS. JS typically shows milder vermian hypoplasia without encephalocele. Importantly, MKS with Dandy‒Walker complex CNS abnormalities may mimic JS (21, 36). (3) Isolated partial vermis hypoplasia (IPVH): MRI reveals an isolated reduction in CV size with a "cleft-like" appearance resembling that in the JSRD but without MTS or associated neurological malformations. (4) Pontocerebellar hypoplasia (PCH): PCH primarily presents in infancy and features progressive pontine atrophy, generalized cerebellar volume loss, and simplified foliation. Diffusion tensor imaging (DTI) reveals reduced fiber bundle density in PCH, which contrasts with the abnormally thickened SCP tracts characteristic of JSRD (30, 35).

**Table 1:** 

Differential Diagnosis	Key MRI Features	Differentiation from JS
Dandy-Walker malformation (DWM)	PF cyst, CV agenesis/hypoplasia, dilated 4V communicating with CM	No SCP thickening, distinct from MTS
Meckel-Gruber Syndrome (MKS)	Occipital encephalocele, obstructive hydrocephalus, cystic renal dysplasia	No MTS, more severe PF anomalies
Isolated Partial Vermis Hypoplasia (IPVH)	Isolated CV hypoplasia with cleft-like appearance	No MTS or other malformations
Pontocerebellar Hypoplasia (PCH)	Progressive pontine atrophy, cerebellar volume loss, simplified foliation	Reduced fiber density on DTI vs. thickened SCP in JS

In our case, we present the first documented instance of prenatal JSRD diagnosed in twins. Fetal MRI evaluation provides critical diagnostic value for the diagnosis, enabling informed prenatal counseling and timely multidisciplinary management at an early stage. The concordant presentation in both dizygotic twins provides compelling evidence for the genetic etiology and underscores the importance of comprehensive fetal assessment in twin pregnancies when an anomaly is detected in one fetus. Our study has certain limitations. Most notably, functional validation assays for the identified *TMEM67* variants were not performed. Therefore, our diagnosis of Joubert syndrome relies on the compelling integration of consistent clinical presentation, the pathognomonic prenatal imaging finding of the molar tooth sign, and the molecular genetic data indicating a compound heterozygous state of two variants in trans.

## 4 Conclusions

In conclusion, Joubert syndrome and related disorders (JSRDs) are congenital anomalies with a high risk of family recurrence. Prenatal diagnosis necessitates a multidisciplinary strategy that integrates clinical, radiological, and genetic insights. When prenatal US reveals fetal posterior fossa morphological abnormalities, fetal MRI should be routinely employed to confirm the diagnosis and evaluate associated neural structural defects. Prenatal MRI can also be combined with genetic testing to optimize treatment decisions, genetic counseling, and prognosis evaluation (37).

## Data Availability

The original contributions presented in the study are included in the article/Supplementary Material, further inquiries can be directed to the corresponding author.

## References

[1] Joubert M Eisenring JJ Robb JP Andermann F. Familial agenesis of the cerebellar vermis: a syndrome of episodic hyperpnea, abnormal eye movements, ataxia, and retardation. 1969. J Child Neurol. (1999) 14(9):554–64. 10.1177/088307389901400902 10488899

[2] Alhashimi I Zoghoul S Khalil SK Yousif ZB Jumah A Alkailani Y. Neuroimaging characteristics as diagnostic tools in joubert syndrome and related disorders: a case report and literature review. Cureus. (2024) 16(9):e69872. 10.7759/cureus.69872 39435230 PMC11493382

[3] Zhu L Xie L. Prenatal diagnosis of Joubert syndrome: a case report and literature review. Medicine (Baltimore). (2017) 96(51):e8626. 10.1097/md.0000000000008626 29390414 PMC5758116

[4] Parisi MA Doherty D Chance PF Glass IA. Joubert syndrome (and related disorders) (OMIM 213300). Eur J Hum Genet. (2007) 15(5):511–21. 10.1038/sj.ejhg.5201648 17377524

[5] Gana S Serpieri V Valente EM. Genotype-phenotype correlates in Joubert syndrome: a review. Am J Med Genet C Semin Med Genet. (2022) 190(1):72–88. 10.1002/ajmg.c.31963 35238134 PMC9314610

[6] Iskender CT Tarım E Alkan O. Joubert syndrome and related disorders, prenatal diagnosis with ultrasound and magnetic resonance imaging. J Turk Ger Gynecol Assoc. (2011) 13(2):135–8. 10.5152/jtgga.2011.75 PMC3939136 24592023

[7] Huang L-X Lu X-G Liu J-X Xu L Shang N Guo L Case report and a brief review: analysis and challenges of prenatal imaging phenotypes and genotypes in joubert syndrome. Front Genet. (2022) 13:1038274. 10.3389/fgene.2022.1038274 36468023 PMC9715754

[8] Wüest A Surbek D Wiest R Weisstanner C Bonel H Steinlin M Enlarged posterior fossa on prenatal imaging: differential diagnosis, associated anomalies and postnatal outcome. Acta Obstet Gynecol Scand. (2017) 96(7):837–43. 10.1111/aogs.13131 28295149

[9] Poretti A Huisman TA Scheer I Boltshauser E. Joubert syndrome and related disorders: spectrum of neuroimaging findings in 75 patients. AJNR Am J Neuroradiol. (2011) 32(8):1459–63. 10.3174/ajnr.A2517 21680654 PMC7964342

[10] Hatab MR Kamourieh SW Twickler DM. MR volume of the fetal cerebellum in relation to growth. J Magn Reson Imaging. (2008) 27(4):840–5. 10.1002/jmri.21290 18302203

[11] Chen SC Simon EM Haselgrove JC Bilaniuk LT Sutton LN Johnson MP Fetal posterior fossa volume: assessment with MR imaging. Radiology. (2006) 238(3):997–1003. 10.1148/radiol.2383041283 16505396

[12] Vatansever D Kyriakopoulou V Allsop JM Fox M Chew A Hajnal JV Multidimensional analysis of fetal posterior fossa in health and disease. Cerebellum. (2013) 12(5):632–44. 10.1007/s12311-013-0470-2 23553467

[13] Prayer D Malinger G De Catte L De Keersmaecker B Gonçalves LF Kasprian G ISUOG practice guidelines (updated): performance of fetal magnetic resonance imaging. Ultrasound Obstet Gynecol. (2023) 61(2):278–87. 10.1002/uog.26129 36722431 PMC10107509

[14] Valente EM Brancati F Dallapiccola B. Genotypes and phenotypes of Joubert syndrome and related disorders. Eur J Med Genet. (2008) 51(1):1–23. 10.1016/j.ejmg.2007.11.003 18164675

[15] Volpe P De Robertis V Volpe G Boito S Fanelli T Olivieri C Position of the choroid plexus of the fourth ventricle in first- and second-trimester fetuses: a novel approach to early diagnosis of cystic posterior fossa anomalies. Ultrasound Obstet Gynecol. (2021) 58(4):568–75. 10.1002/uog.23651 33847428

[16] Dempsey JC Phelps IG Bachmann-Gagescu R Glass IA Tully HM Doherty D. Mortality in Joubert syndrome. Am J Med Genet A. (2017) 173(5):1237–42. 10.1002/ajmg.a.38158 28371402

[17] Komatsu Y Suzuki T Tsurusaki Y Miyake N Matsumoto N Yan K. TMEM67 Mutations found in a case of Joubert syndrome with renal hypodysplasia. CEN Case Rep. (2016) 5(2):137–40. 10.1007/s13730-015-0210-1 28508964 PMC5413751

[18] Richards S Aziz N Bale S Bick D Das S Gastier-Foster J Standards and guidelines for the interpretation of sequence variants: a joint consensus recommendation of the American college of medical genetics and genomics and the association for molecular pathology. Genet Med. (2015) 17(5):405–24. 10.1038/gim.2015.30 25741868 PMC4544753

[19] Lord J McMullan DJ Eberhardt RY Rinck G Hamilton SJ Quinlan-Jones E Prenatal exome sequencing analysis in fetal structural anomalies detected by ultrasonography (PAGE): a cohort study. Lancet. (2019) 393(10173):747–57. 10.1016/s0140-6736(18)31940-8 30712880 PMC6386638

[20] Wang S Li M Chen G Chen Z Lei K Ökten Z Joubert syndrome 26 protein enforces compartmentalized motility of a ciliary kinesin. Proc Natl Acad Sci U S A. (2025) 122(47):e2504374122. 10.1073/pnas.2504374122 41264249 PMC12663925

[21] Stembalska A Rydzanicz M Pollak A Kostrzewa G Stawinski P Biela M Prenatal versus postnatal diagnosis of Meckel-Gruber and Joubert syndrome in patients with TMEM67 mutations. Genes (Basel). (2021) 12(7):1078. 10.3390/genes12071078 34356094 PMC8304314

[22] Khalil A Rodgers M Baschat A Bhide A Gratacos E Hecher K ISUOG practice guidelines: role of ultrasound in twin pregnancy. Ultrasound Obstet Gynecol. (2016) 47(2):247–63. 10.1002/uog.15821 26577371

[23] Grody WW Thompson BH Gregg AR Bean LH Monaghan KG Schneider A ACMG position statement on prenatal/preconception expanded carrier screening. Genet Med. (2013) 15(6):482–3. 10.1038/gim.2013.47 23619275

[24] Pugash D Oh T Godwin K Robinson AJ Byrne A Van Allen MI Sonographic 'molar tooth' sign in the diagnosis of Joubert syndrome. Ultrasound Obstet Gynecol. (2011) 38(5):598–602. 10.1002/uog.8979 21370303

[25] Doherty D Glass IA Siebert JR Strouse PJ Parisi MA Shaw DWW Prenatal diagnosis in pregnancies at risk for Joubert syndrome by ultrasound and MRI. Prenat Diagn. (2005) 25(6):442–7. 10.1002/pd.1145 15966043

[26] Cater SW Boyd BK Ghate SV. Abnormalities of the fetal central nervous system: prenatal US diagnosis with postnatal correlation. Radiographics. (2020) 40(5):1458–72. 10.1148/rg.2020200034 32706613

[27] Bachmann-Gagescu R Dempsey JC Bulgheroni S Chen ML D'Arrigo S Glass IA Healthcare recommendations for Joubert syndrome. Am J Med Genet A. (2020) 182(1):229–49. 10.1002/ajmg.a.61399 31710777 PMC7679947

[28] Hsu CC Kwan GN Bhuta S. High-Resolution diffusion tensor imaging and tractography in Joubert syndrome: beyond molar tooth sign. Pediatr Neurol. (2015) 53(1):47–52. 10.1016/j.pediatrneurol.2015.02.027 25890865

[29] Lee S-K Kim DI Kim J Kim DJ Kim HD Kim DS Diffusion-tensor MR imaging and fiber tractography: a new method of describing aberrant fiber connections in developmental CNS anomalies. Radiographics. (2005) 25(1):53–65. 10.1148/rg.251045085 15653586

[30] Akbari K Fellner CM Flöry D Fellner FA. CT And MR imaging findings in the Joubert syndrome, a “ciliopathy”. J Biomed Sci Eng. (2015) 8(10):695. 10.4236/jbise.2015.810066

[31] Glass IA Dempsey JC Parisi M Doherty D. Joubert syndrome. In: Adam MP Feldman J Mirzaa GM Pagon RA Wallace SE Amemiya A, editors. GeneReviews®. Seattle: University of Washington (1993). p. 5. 20301500

[32] Quarello E Molho M Garel C Couture A Legac MP Moutard ML Prenatal abnormal features of the fourth ventricle in Joubert syndrome and related disorders. Ultrasound Obstet Gynecol. (2014) 43(2):227–32. 10.1002/uog.12567 23868831

[33] Maria BL Bozorgmanesh A Kimmel KN Theriaque D Quisling RG. Quantitative assessment of brainstem development in Joubert syndrome and Dandy-Walker syndrome. J Child Neurol. (2001) 16(10):751–8. 10.1177/088307380101601008 11669349

[34] Jissendi-Tchofo P Doherty D McGillivray G Hevner R Shaw D Ishak G Pontine tegmental cap dysplasia: MR imaging and diffusion tensor imaging features of impaired axonal navigation. AJNR Am J Neuroradiol. (2009) 30(1):113–9. 10.3174/ajnr.A1305 18842761 PMC3919876

[35] Poretti A Boltshauser E Loenneker T Valente EM Brancati F Il'Yasov K Diffusion tensor imaging in Joubert syndrome. AJNR Am J Neuroradiol.. (2007) 28(10):1929–33. 10.3174/ajnr.A0703 17898198 PMC8134246

[36] Srivastava S Manisha R Dwivedi A Agarwal H Saxena D Agrawal V Meckel Gruber and Joubert syndrome diagnosed prenatally: allelism between the two ciliopathies, complexities of mutation types and digenic inheritance. Fetal Pediatr Pathol. (2022) 41(6):1041–51. 10.1080/15513815.2021.2007434 34821546

[37] Torres JA Escobar BF Martinez JG Martínez RA Cortez FP. Radiological features of joubert syndrome and clinical case presentation. Radiol Case Rep. (2024) 19(10):4167–72. 10.1016/j.radcr.2024.06.065] 39101024 PMC11293500

